# For a science of layered mechanisms: beyond laws, statistics, and correlations

**DOI:** 10.3389/fpsyg.2014.00536

**Published:** 2014-06-03

**Authors:** Cristiano Castelfranchi

**Affiliations:** Institute of Cognitive Sciences and Technologies, Consiglio Nazionale delle RicercheRome, Italy

**Keywords:** reductionism, cognitive architecture, emergence, intentions, functions, computer modeling and simulation, proximate causes

## Abstract

Two general claims are made in this work. First, we need several different *layers* of “theory,” in particular for understanding human behavior. These layers should concern: the cognitive (mental) representations and mechanisms; the neural underlying processes; the evolutionary history and adaptive functions of our cognition and behaviors; the emergent and complex social structures and dynamics, their relation and feedbacks on individual minds and behaviors, and the relationship between internal regulating goals and the external functions/roles of our conduct; the historical and cultural mechanisms shaping our minds and behaviors; the developmental paths. Second, we do not just need “predictions” and “laws” but also “explanations”; that is, we need to identify the mechanisms producing (here-and-now, or diachronically) a given phenomenon. “Laws” are not enough; they are simply descriptive and predictive; we need the “why” and “how.” Correlations are not enough (and they are frequently misleading). We need computational models of the processes postulated in our theories^1^.

## THE NEED FOR EXPLANATION: MAIN ISSUES

We do not just need a “pluralistic” approach (as radically inter-disciplinary) but a “layered” theory of our “objects” (Dale, 2008). We need (at least) six layers and axes of theory; not just predictions but explanations, that is, *we need to identify the explicit definition/understanding of the mechanisms producing* (here-and-now or diachronically) *a given phenomenon*. In particular for human behavior we need:

(A) *Modeling the cognitive (mental) mechanisms producing and governing (controlling) our behavior*. That is, we have to explain a given behavior with its “proximate” causes: micro-processes, irreducible to the vocabulary (ontology) of neuro-processes.
(B) *The neural and body implementation of psychological representations and processes*. We should know not only *where* they are located in the brain, but the brain micro-mechanisms and emergent cognitive processes, and *why* they work there.
(C) *The biological evolution of our behavior* (and its “causes”: adaptive functions, niche, environmental constraints) and of the mental (cognitive, motivational, affective) mechanisms selected for governing it. Without understanding the “origin,” the diachronic causes, we cannot fully explain a phenomenon.
(C1) This requires the understanding of the relation between our genes and our behavior; the dispositions selected by evolution, and how they influence our mental processes and behavior, and how these inherited “programs” interact with experience, learning, and culture.
(C2) This also requires the understanding of the relations between the two kinds of teleology that impinge on us: the internal goals regulating/controlling our action vs. the external functions of our conduct.
(D) *The emergent, collective, self-organizing effects of our behaviors, and their mechanisms and dynamics*; how complexity determines the “social orders.” An analytic and dynamic theory of the “invisible hand.” Otherwise, we cannot understand societies, etc., as well as the relation between emergent collective phenomena and our intentions and mental representations: *How* is it possible that we “pursue” ends that we do not are aware of and are not among our intentions? We also need to explain how the emergent structure/order feedbacks into, and shapes, our minds and behaviors: not just the “emergence” but also the “immergence” processes.
(E) The historical and cultural evolution – its mechanisms, not just its description and narration – shaping our minds and behaviors, through learning, practices and technologies, norms, and so on. The cultural evolution is not less relevant than the biological one for understanding why we are as we are; and our historical and cultural differences.
(F) We need the modeling of developmental processes, also because some causes of our adult behavior can be found in our personal and relational development (Developmental Cognitive Sciences).

We needs at least all these layers and perspectives (diachronic and evolutional) for *explaining* our behaviors. Laws are not enough. They are simply descriptive and predictive; we need the “why,” the “how” (see Cognitive Mechanisms Producing and Controlling Our Behavior & Computational Science for Reconciling “Emergence” with “Cognition”6). Of course, the need for explaining (not just predicting and describing) with the underlying devices the observed and observable phenomena, is particularly crucial for the cognitive and behavioral sciences, where the observable phenomena are due to unobservable postulated variables into the minds. But – in my view – is not valid only for human sciences. To be less schematic and more correct, let me say that there are in the natural science “laws” that do really explain in terms of causal underlying “mechanisms” producing the phenomenon and its dynamics. This is the case – in my view – for example of mutation and selection mechanisms explaining Darwinian evolution; although also these mechanisms and laws have to be explained at their micro layer in terms of genes and DNA mechanisms. However, many important “laws” in natural science are not really “explanatory” of the “why” and “how,” of the mechanism. For example, the most famous natural law, Newton’s gravity, is more descriptive than based on the explanation of the (micro) mechanisms producing/causing attraction. We are still in search of the real causal explanation: the “graviton.” At the higher level of course that law predicts but also causally “explain” why something (pears) falls to the ground (from the trees).

No brain map too is enough: it is just cartography, descriptive rather than explicative (see The Neural Implementation of Psychological Representations and Processes).

Correlations are not enough and they are frequently misleading (Concluding Remarks). Theories should be complemented by *models* of the processes that produce and control people’s behavior.

(G) This is why a crucial revolution in the behavioral sciences is and will be the computational modeling: the radical “operational” approach. There is no alternative to this especially if one has to model the process at a given micro-layer, and the processes at the macro-layer, and also the emergent (bottom-up) and the immergent (top-down) feedbacks, and how all this works.

I will only focus on issues (A) (C) (D) (G), and in a quite schematic and assertive way also on (B). However, there is a coherence between the central claim of section 5 (point D) on “social” theory (social action and minds are crucial but not enough; we need a theory of the self-organizing macro social order and of its feedback at the micro level) and the seemingly far polemic on (B; brain and mind): layered view is needed because reality is a recursive multi-layered “emergent” complex system: not only we have emergence (and self-organization) from individual to collective, but also from micro neuro-processes and macro functions in brain, and from brain to mental activities, and from cognitive micro-constituents (like beliefs or goals) to complex mental states “gestalts,” like an intention (Knowledge–Motivation Commerce), an expectation, or an emotions like hope (Miceli and Castelfranchi, 2014); and so on (A Layered Science for a Layered World).

## COGNITIVE MECHANISMS PRODUCING AND CONTROLLING OUR BEHAVIOR

As we said (A), to *explain* a given behavior we need to identify its “proximate” cognitive causes -underlying processes that are irreducible to neuro-processes: representational and functional. Of course, also neural processes are “representational” and based on “functional” notions (like “activation,” “inhibition,” “connection,” etc.), but at a lower micro-level.

In particular, what is needed is a theory of how our behavior is under a “control device”; its mainly goal-governed nature, and how motivations are organized and processed. This is the weakest part of psychology: we know everything about knowledge processing and organization (step by step, all the chapters of a handbook of Cognitive Psychology), but we know very little about motivation and its processing. In particular we should model how our goals (used here as a general term for internal motivational representations during the cybernetic cycle (Miller et al., 1960); including wishes, desires, concerns, intentions, and so on) are processed (activated, chosen, preferred, planned) on the basis of our beliefs, and how we acquire and integrate or revise them. The central mechanism of mind is the “commerce” between goals and beliefs (the two basic families of mental representations). Of course also other “mechanisms” are there; simple reflexes, conditioned reactions, habits, routines, scripts, and so on.

To exemplify the kind of cognitive architecture we should model, let us focus on the last stage of the processing of a goal and on its final package, which regulates *intentional* action (as a specific kind of “behavior”).

### KNOWLEDGE–MOTIVATION COMMERCE

Intentions are those goals that *actually drive our voluntary actions or are ready/prepared to drive them*. They are not another primitive (like in BDI model inspired by Bratman’s theory, e.g., Rao and Georgeff, 1995), a different mental object with respect to goals. They are just a kind of goal: the final stage of a successful goal-processing, which also includes “desires” in the broad sense, with very specific and relevant properties (see also Castelfranchi and Paglieri, 2007). Let’s remark that the creation of two distinct “primitives,” basic independent notions/objects (“desires” vs. “intentions”) is in part due to the wrong choice of adopting (also in accordance with common sense) “desires” as the basic motivational category and source. We criticize this reductive move, and introduce a more general and basic (and not fully common sense) teleonomic notion of “goal.” This notion also favors a better unification of goal kinds and a better theory of their structural and dynamic relationships.

**FIGURE 1:**
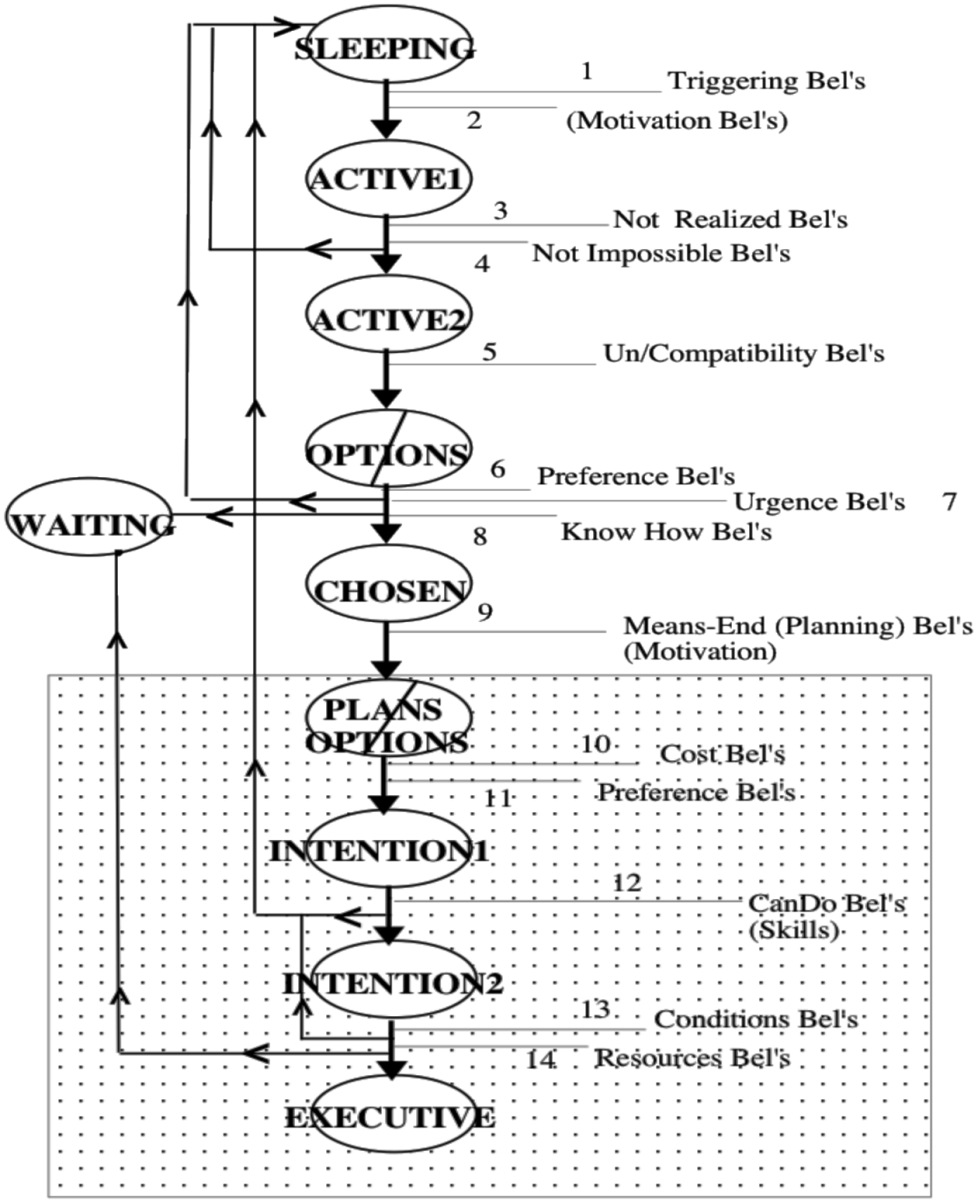
**Beliefs in Goal-Processing (Castelfranchi and Paglieri, 2007)**.

In a nutshell (**Figure 1**), in our model an *intention* is a goal that:

(1) Has been activated (by a physiological stimulus, an impulse, or an emotion, or just by a new belief) and processed.
(2) Has been evaluated (beliefs) as not impossible, and not self-realizing or already realized by another agent, and thus *up to us*: we have to act in order to achieve it. An intention is always the intention to “do something” (including inactions). We cannot really have intentions about the actions of other autonomous agents. When we say something like “I have the intention that John goes to Naples” what we actually mean is “I have the intention *to bring it about that* John goes to Naples.”
(3) Has been chosen against other possible active and conflicting goals, on the basis of an evaluation (beliefs) of the outcomes and possible costs and we have “decided” to pursue it as *preferable* (greater expected value) to its competitors.
(4) Is consistent with other intentions of ours; a simple goal can be contradictory, inconsistent with other goals, but, once it is chosen, it becomes an intention and has to be coherent with the other intentions (beliefs about action conditions, resources, and compatibility in the word; Castelfranchi and Paglieri, 2007). Decision-making serves precisely the function of selecting those goals that are feasible and coherent with each other, and allocating resources and planning one’s actual behavior.
(5) Implies the agent’s beliefs that she knows (or will/can know) how to achieve it, that she is able to perform the needed actions, and that there are or will be the needed conditions for the intention’s realization; at least the agent believes that she will be able and in condition to “try.”
(6) Being “chosen” implies a *commitment* with ourselves, a mortgage on our future decisions; intentions have priority over new possible competing goals, and are more persistent than the latter (Bratman, 1987).
(7) Is “planned”; we allocate/reserve some resources (means, time, etc.) for it; and we have formulated or decided to formulate a plan consisting of the actions to be performed in order to achieve it. An intention is essentially a two-layer structure: (a) the “intention that,” the *aim,* that is, the original goal (for example, to be in Naples tomorrow); (b) the “intention to do,” the sub-goals, the planned executive actions (to go to the station, buy the ticket, take the train, etc.). There is no intention without (more or less) specified actions to be performed, and there is no intention without a motivating outcome of such action(s).
(8) Thus an intention is the final product of a successful goal-processing that leads to a goal-driven behavior.

Thus “intention” is not a simple mental object (although outcome of a complex process); it is a *complex configuration* with its *anatomy*: of supporting beliefs and of goals in a means-end relation, and with an impendent commitment.

After a decision to act, an intention is already there even if the concrete actions are not fully specified or are not yet in execution, because some condition for their execution is not currently available. Intentions can be found in two stages:

(a) *Intention “in action,”* that is, guiding the executive intentional action;
(b) *Intention “in agenda”* (“future directed,” those more central to the theories of Bratman, Searle, and other), that is, already planned and waiting for some lacking condition for their execution: time, money, skills, etc. For example, I may have the intention to go to Capri next Easter (the implementation of my “desire” of spending Easter in Capri), but now is February 17, and I am not going to Capri or doing anything for that; I have just decided to do so at the right moment; it is already in my “agenda” and binds my resources and future decisions.

I would also say that an “intention” is “conscious,” we are aware of our intentions and we “deliberate” about them; however, the problem of unconscious goal-driven behavior is open and quite complex (see Bargh et al., 2001).

During the several steps of its processing, an intention – and its original goal – is supported by those beliefs (on the past, the present, or the future) that are filtering and supporting it. Whenever one of such beliefs changes, there may be a problem for the supported goal, which may be either put in a “waiting room” or abandoned as impossible, already achieved, no longer interesting (because another is deemed to be preferable.), etc. When dealing with *cognitive* agents, in order to change their behavior we have to change their goals (and thus their intentions), but in order to change their goals we have to change their beliefs.

## THE NEURAL IMPLEMENTATION OF PSYCHOLOGICAL REPRESENTATIONS AND PROCESSES

A neuroscience of human behavior should *in primis* be the neural modeling of cognitive *mechanisms* and *processes* postulated by the Cognitive Sciences.

Neuroscience shouldn’t give us a brain “cartography” of behaviors and feelings: cartography has never been a “science” (just a technique); it explains nothing, it is just description. What we need is the brain/body *implementation* of specific functions and models of elaboration of representations, which determine our conduct.

### THE NEUROSIS OF BEHAVIORAL SCIENCES

Neuroscientists shouldn’t try to “skip” psychology and its information-processing models of structures and manipulations, for directly connecting brain with behavior (neuro-economics, neuro-aesthetics, neuro-ethics, neuro-politics,…). On the contrary they should take the procedural (possibly computational) models of the cognitive sciences and find their neural grounding or – if this proves unfeasible – change them. In fact, a cognitive model that is not grounded in our brain and somatic processes is just wrong, unacceptable. And – on the other side – psychology should provide models of proximate processes; not just correlational “theories,” which say nothing on the *mechanisms*.

Actually, there is a minority of approaches that look me rather different and going in a much more promising direction: to analyze the specific “implementation” of *psychological processes and model* in brain functions, processes, and “goals” (Goals vs. Pseudo-goals). Aimed to materialize (they say “embody”) cognitive functions in their physical and informational substrate. A very good prototype is for example (Friston et al., 2013) work on the *physical* dynamics in the brain that implement the functions and psychological mechanisms (confidence, expected utility, attainability, inferences, etc.) postulated in decision-making processes.

Nevertheless, in my view, the shortcut temptation I’m pointing on is there, is dominant, and is a misleasing perspective.

The problem is: will neurosciences be able to distinguish, for example, between mere anticipation of benefits or costs, where the expected (and perhaps desirable) result is just predicted, and when this anticipatory representation plays the functional role of (achievement or avoidance) goal? Moreover, expected outcomes that we predict and appreciate/desire are not the same of the expected outcomes that *motivate* our actions: that is, not just additional positive results but those that are *necessary* and *sufficient* for acting.

This is a really crucial distinction (that must be neurologically founded) for a theory of human conduct. Without that it would/will be impossible to distinguish, for example, between:

– Utility-driven vs. value- or norm-driven behavior; or between
– True “altruistic” and non-altruistic pro-social actions.

In fact – in psychological terms – the altruistic nature of an action only depends on the mind-set of the agent. Considering an act as “altruistic” implies a “judgment on mere intent^2^.” “Altruistic” is a *subjective* notion, relative to the underlying mental representations (especially the motivational ones); it is not – in human beings – just a behavioral and objective notion. It is not enough that a given conduct is beneficial for Y and costly for X (the agent); even if the benefit is intentional. It is necessary to ascribe to X the motivation to favor Y’s wellbeing, rather than some possible expected (external or internal) reward. Thus it would be insufficient to find that these conducts are associated with the activation of a brain area which is related to a “predictive” or anticipatory activity, or to pro-social emotions.

Another example is offered by the neural version of “trust.” As Fehr writes: “the rationale for the experiment originates in evidence indicating that oxytocin plays a key role in certain pro-social approach behaviors in non-human mammals. (…) Based on the animal literature, Kosfeld et al. (2005), hypothesized that oxytocin might cause humans to exhibit more behavioral trust as measured in the trust game” (Fehr, 2009). In these experiments they also show how oxytocin has *a specific effect on social behavior* because it differently impacts on the trustor and the trustee (only in the first case there is a positive influence). In addition, it is also shown that the trustor’s sensitivity to risk is not reduced as a general behavior but it depends on the partner nature (human versus non-human). These are no doubt interesting data. However, the multidimensional and very articulated notion of trust should not be reduced to a generic pro-social attitude and to a particular chemical response or the mere activation of a given brain area. Trust is not a simple, vague, and unitary notion and disposition; it is made of (rather complex) evaluations, expectations, attributions, decisions to rely, sentiments. It should be a componential and analytical psychological model of trust to *drive* the neural research rather than searching for a simplistic and direct solution, just localistic and correlational (Castelfranchi, 2009).

Analogously, consider norm compliance: will neurosciences be able to distinguish the explicit understanding and processing of a norm and the decision (and reasons) to comply with it, from a merely habitual conforming conduct? And in motivated obedience will neurosciences distinguish between just expected possible sanctions and a decision “motivated” by that avoidance? Will they reduce norms just to the activation of feared punishments or of inhibitory responses? Psychologically speaking, these are very different processes, with quite different socio-political implications.

Finally, we should accept the idea that, in social “games” and scripts, part of the mental attitudes we ascribe to others are not “materially” in their brains. Also mind is an “as if,” an “institutional construct.” We ascribe certain contents (knowledge, goals,…) to others and we act on such as basis, *as if* they were materially there, and this works in our “social pretending”: we give them a real, pragmatic, effect, like when we turn pieces of paper into money, by accepting and using them as such (Castelfranchi, 2013). For example, for sure you “know” that 126 + 32 = 158, or you “know” that Athens is not the capital of Italy, but do you really have this knowledge written in a file of your brain? Not at all! Only after you derived it, not before; however, you implicitly and potentially “know” that and I know that you know and interact with you on such a basis.

Mind is not independent on brain, and in general on a material support of “information processing.” Mind is what the brain does but not at its micro level; at the level of macro-functions and complex object (representations). However, “mind” is not only what the brain does. Not only because we might have minds “embodied” in other “machineries” or supports (also at the distributed social interaction level); but because mind is also an “intentional stance” creation, attribution, ascription in order to explain, predict the behavior, and interact with. It is a crucial “instititional” object, even independent of its brain content, like the “value” of money, no longer dependent on gold.

### ARBITRARY ASSUMPTIONS IN MIND-BRAIN-BEHAVIOR RELATION

The current views on the relation between psychological processes and their neuro-chemical substratum often betray some questionable assumptions. For example, whereas it is very reasonable to suppose that psychological and support interventions may have an impact on cerebral regulation, at a biological level, this by no means implies that the *origin* of the problem was biological, in terms of a biochemical or neural malfunction.

Mental representations and psychological processes are *per se* IN our brain (if not, where else might they be found?!) and are processes OF our brain. Every construction, acquisition, or elaboration of them just is a neural pattern/process in which our mind materially consists and is implemented^3^.

However, to acknowledge this truism does not mean that research at the psychological layer has no longer need to be conducted: psychological notions and models should be neurologically grounded, not “eliminated” (Computational Science for Reconciling “Emergence” with “Cognition”); moreover, one should be aware of the (not just theoretical) risks of biological reductionism and their impact on public opinion; consider for instance the growing tendency of psychiatry to adopt (in theory and in practice) a bio-pharmacological approach, and its problematic consequences at the scientific, social, political, and ethical levels.

Actually, there is a *non sequitur* between the (obvious) idea that dysfunctional/psychopathological (and recovery) processes are *brain processes* and

(i) the assumption that *therefore* their cause *must* be a brain damage, a neural or biochemical dysfunction, a neural *disease*;
(ii) the assumption that *therefore* [even independently of claim (i)] the intervention must necessarily and *directly* be on the brain and its functioning.

To think something is a new state of our brain; to learn something is to modify our brain; to relearn, adjust previous learning, is to modify our brain again. There might have been (for several concurrent factors: internal and external, experiential, relational) a *dysfunctional* learning, dysfunctional thoughts, and the challenge is – through new cognitive and affective experiences and mental elaborations – restructuring the learned representations and processes.

Any change in our conduct or attitudes is/presupposes a change in our minds; any change in our minds is/ presupposes a change in our brains (and bodies). Our brain has been materially “written” by our conduct. In therapeutic, educational or rehabilitation interventions the challenge is to preserve this route, and this view. For changing our brain we do not need to directly act on our brain. Similarly, for producing water we do not need (and it is even worst) to join oxygen and hydrogenous; or for changing genes regulation not necessarily we manipulate genes (epigenetics).

## TWO TELEOLOGIES IMPINGING ON HUMAN BEHAVIOR

As for the biological evolution issue (point C), let me just consider a crucial theoretical issue (C2), which is often neglected or mistreated: *the relation between the two kinds of teleology that impinge on us: the internal goals regulating/controlling our action vs. the external functions of our conduct.*

In modern science there are two well-defined teleological frames and notions:

The one provided by *evolutionary approaches*, where it is standard (and correct) to talk in terms of *functions* (adaptive) value, being for something, having a certain finality/end, providing some advantage, etc. In this context “goal” (end, function, finality, etc.) means the “effect” (outcome) that has selected/reproduced and maintained a certain feature or behavior – originally just an accidental effect, an effect among many others, but later, thanks to the loop and positive feedback on its own causes (that is, on the feature or behavior producing it) no longer a mere effect but the *function*, the purpose of that feature, what makes it useful and justifies its reproduction.

• The one provided by *cybernetic control theory* and its postulated cycle, representations, and functions, in which the agent is able to adjust the world through goal-directed behavior, and to maintain a given desired state of the world (homeostasis).
• Actually, there might be a third teleological/finalistic notion used in several sciences (from medicine to social sciences): the notion of a *function* of X as a *role*, a functional component, an “organ” of a global “system.” For example, the function of the heart, or of the kidneys, in our body; or the function of families (or of education or of norms) in a society; or the *function* of a given office in an organization; etc. However, this functionalist and systemic notion has never been well defined and has elicited a lot of problems and criticisms. My view is that this finalistic view is correct, but it is reducible to, and derived from, the previous two kinds of teleology. The organs are either the result of an evolutionary selection – in that they contribute to the fitness and reproduction (maintenance) of that organism – or there is a project, a design, that is, a complex goal in someone else’s mind, which imposes particular sub-goals on its parts, components, and tools. Or both.

A serious problem for a (future) science of goals is the fact that these two fundamental teleological notions/mechanisms have never been unified:

(i) Neither conceptually, by looking for a common definition, a conceptual common kernel (for example, in terms of circular causality, feedback, etc.). Do we have and is it possible to have a general, unique notion of “goal” with two sub-kinds (functions vs. psychological goals)?
(ii) Nor by solving the problem of the interaction between the two coexisting forms of finality.

This constitutes a serious obstacle, and reveals a real ignorance gap in contemporary science^4^. For example, as for issue (i), without the aforementioned conceptual unification we cannot have a unitary theory of communication – or a theory of cooperation, of sociality, etc. – in animal and humans. What today are presented as unified theories are just a trick; in fact, those notions – which necessarily require a goal (for example, communication doesn’t just require a “reader,” it requires a “sender”: the information is given on purpose to the receiver/addressee) – are defined in terms of adaptive *functions* when applied to simple animals (like insects), whereas in humans are defined in *intentional* terms. Thus there is no unified notion (and theory) of communication, in that we do not know the common kernel between a *functional* device and an *intentional* device.

Point (ii) is no less problematic. *What is the relationship between the internally represented goals (motivations, and concrete objectives) of an agent regulating its behaviors from the inside, and the adaptive functions that have selected that agent and its behaviors*?

**FIGURE 2:**
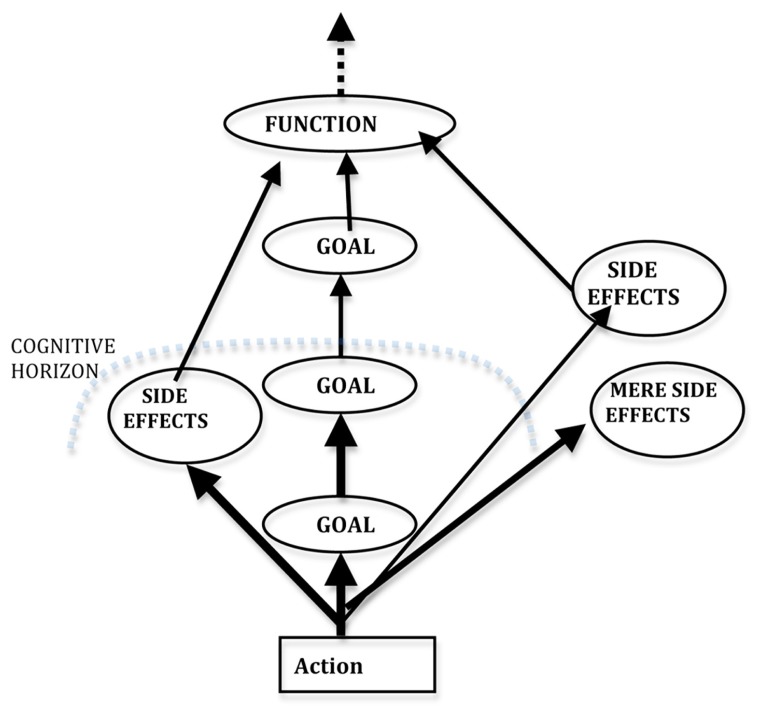
**Mental Goals and possible functions**.

Usually, in purposive, goal-driven agents/systems, the *function* of their conduct, the adaptive result that has to be guaranteed, is *not* internally represented and psychologically pursued; it is not understood and foreseen (**Figure 2**). Of course not all the foreseen outcomes or all the side effects have a *function*.

The internal motivations (and whatever solutions and instrumental goals they generate) may just be sub-goals of the “external” goals of the behavior, of its functions; they are just “cognitive mediators” of the (biological or social) *functions* that would be non-representable and mentally non-computable. For example, only very recently we have discovered why we have to eat, the real functions/effects of our food in our organisms (proteins, carbohydrates, vitamins, etc.); and very few people eat in view of such effects. We eat for hunger or for pleasure or for habit. Analogously, we do not usually make courtship and sex in view of reproduction; we are driven by other internal motives.

Because our behavior may respond to two kinds of teleology – *internal*, driving *goals* (control theory model) vs. *external* selective *functions*, either biological or social (Castelfranchi, 2001) – this is why there might be *conflicts* between one’s internal goal and the *function* of one’s action/behavior – also considering that we do not necessarily understand and thus intentionally pursue our biological or social functions. We may even act against the functions of our behavior. We may even cut the adaptive connection between our motives and their original functions, for example by deciding to have sex without inseminating or without establishing/maintaining any friendly/affective/supportive relation with our mate. As for social functions, an example of a conflict between our goals and our *role function* could be the goal that B be condemned while I’m his defense attorney.

As for social functions and roles in general, we play them (citizen, consumer, father, pedestrian,…) quite blindly; not because they are unconscious, or because just based on reinforcement learning or on mere “habituses” (in Bourdieu’s view; see The Emergent, Collective, Self-Organizing Effects of our Behaviors), but because they are external to our minds. In fact, even our intentional and deliberated actions, evaluated on their (visible and conceivable) consequences, may “pursue” collective (good or bad) external “ends” (Castelfranchi, 2001).

For example, if we realize how marketing induces “needs,” and deceives and manipulates us, we couldn’t play well our most crucial “role” in/for society: the role of “consumers”!

Social functions are *parasitic* to cognition: they establish and maintain themselves *thanks to and through agents’ mental representations but not as mental representations: i.e., without being necessarily known or at least intended.* “By pursuing his own interest he [the individual] frequently promotes that of the society more effectually than when he really intends to promote it.” (Adam Smith; last sentence of cited paragraph in section “The Emergent, Collective, Self-Organizing Effects of our Behaviors.”) However, it is possible, and even frequent, that – following our personal motives – we play our roles in contradiction with the mission and collective utility of our social function.

### GOALS VS. PSEUDO-GOALS

It is also very important to disentangle true goals from *pseudo-goals* (Miceli and Castelfranchi, 2012), that is, goals that only seem to be there and to regulate the system and its behavior. However, in fact they are not there as goal mechanisms, they are not represented in the system and “governing” it. They are just functional ways in which the system has been “designed” (by evolution, by learning, by the designer); they are the system’s goal-oriented way of working, its operational rules. For example, a real thermostatic system (thermostat, thermometer, room, radiator, boiler, etc.) has been designed in order to reduce naphtha consumption, heat loss, etc. as much as possible. These are (pseudo)goals of the system, which works also in order to guarantee them; but they are not true cybernetic-goals like the set-point of the thermostat. They are not represented, evaluated, and “pursued” by the system action cycle.

Analogously, our minds have been shaped (by natural selection, or culture and learning) in order to have certain working principles and to guarantee certain functions, which are not explicitly represented and intended. It seems (from our behavior) that we have certain goals, but they are not real goals, only pseudo-goals. This is the case, in our view, of some well-known (and badly misunderstood) finalistic notions, like utility maximization, cognitive coherence, and even pleasure. No doubt, we often choose between different possible goals so as to maximize our expected utility, giving precedence/preference to the greater expected value; that is obvious and adaptive. However, this does not mean that we have “the” goal (the unique and monarchic goal) of maximizing our utility, indifferently to the specific contents and goods. On the contrary, we are moved and motivated by specific, qualitative terminal goals of ours (esteem, sex, power, love, etc.), but the *mechanism* that has to manage them has been designed and works so that it maximizes expected utility.

In the same vein, we maintain coherence among our beliefs, and need to avoid and eliminate contradictions. That is why we can reject certain information and do not believe all the data we get (sometimes even what we directly perceive; “we do not believe our eyes,” literally); the new data must be plausible, credible, integrable, within the context of our preexisting knowledge; otherwise, we have to revise our previous beliefs on the basis of new (credible) data. This coherence maintenance is frequently completely automatic and routinely. We have mechanisms for coherence check and adjustment. We do not usually have any real intention about the coherence of what we believe. Thus, knowledge coherence is a pseudo-goal of ours, not a real meta-goal guiding meta-actions.

### PLEASURE

Similarly, pleasure is not “the” goal of our activity, and the same holds for feeling pleasure (or avoiding feeling pain). “Pleasure” – as a specific and qualitative subjective experience, sensation (not as an empty tautological label for goal satisfaction) – normally is not a goal for us: it is not what we intend to realize/achieve while acting, what move us for performing that behavior. Of course, feeling pleasure or avoiding pain *might* become real goals and intentionally drive our actions: that is basically the mindset of the true hedonist, who acts for pleasure and not for whatever practical consequence his/her action accomplishes. But typically looking for pleasure and avoiding pain are not a unique final goal of ours (another monarchic view of mind and motivation): rather, they act as signals for learning, and they help us learning, among other things, how to generate and evaluate goals.

Those hedonistic philosophies that identify pleasure with motivation, and relate our goal-oriented activity to pleasure motivation, should address the following, evident objections:

i. As a matter of fact, several goals when attained do not give us any pleasure experience; they are just practical results, or consist in the pursuit of (often unpleasant) duties.
ii. If pleasure is so necessary for goal pursuit and motivated activity, why it is not necessary at all in cybernetic models of goal-directed activity and purposive systems? How is it possible to have a clearly finalistic and anticipation-driven mechanism, open to “success” and “failure,” without any pleasure? In other terms, *what is the real function and nature of pleasure in a goal-directed system?* Moreover, pleasure seems to be present in nature (both phylogenetically and ontogenetically) well before mentally goal-directed actions. This also suggests that the function of pleasure has to be different; it does not seem to play the role of a goal.

In my view, pleasure is more related to the notion of “reward,” of “reinforcement” and learning. Pleasure as an internal reward plays two fundamental roles: it attaches some value to some achieved state, which is important when the system can have more than one of such states, possibly in competition with each other; it signals that a given outcome (perhaps accidental) “deserves” to be pursued, is good, has to become a goal (that state, not the pleasure *per se*). In this view, pleasure is a signal and a learning device for goal creation/discovery and for evaluation. It seems very useful in a system endowed with a “generative” goal mechanism, and which needs different kinds of evaluation, more or less intuitive, fast, based on experience or on biological/inherited “preferences,” and not just on reasoning (with its limits, biases, and slowness).

## THE EMERGENT, COLLECTIVE, SELF-ORGANIZING EFFECTS OF OUR BEHAVIORS

That is how complexity determines the “social order.” Our claim on issue (D) is that we need an analytic and dynamic theory of the “invisible hand,” aimed at identifying its underlying mechanisms. Otherwise we cannot understand societies, etc. We also have to understand the relation between the mechanisms regulating the social order and our intentions and mental representations: that is, how – without being understood and explicitly represented – the emergent structure/order feedbacks into and shapes our minds and behaviors; not just the “emergence” but also the “immergence” processes.

The *foundational* issue of the Social Sciences is the micro-macro link, the relation between cognition and individual behavior and social self-organizing phenomena or complex structures and organizations^5^; and institutional actions/phenomena (the two facets of “social order”: the “spontaneous” one and the organized or at least institutionalized one; Tummolini and Castelfranchi, 2006). This is the main reason for the existence of the social sciences, what they have to “explain,” diachronically and synchronically, in its origin and dynamics.

As remarked by Hayek (1996): “This problem [the problem of the unintentional emergence of order and of spontaneous institutions] is in no way specific to economics… *it is without doubt the core problem of the whole of social science.”*

That is also why *Methodological Individualism,* although fundamental or better necessary, is not *sufficient* at all as a framework for explaining social interactions and phenomena (Conte and Castelfranchi, 1995).

Adam Smith’s original formulation of “THE problem” is – to me – much deeper and clearer than Hayek’s formulation.

The great question is how [the individual] *“which does neither, in general, intend to pursue the public interest, nor is aware of the fact that he is pursuing it,* … *is conducted by an invisible hand to promote an end that is not part of his intention”* (Smith, 1976).

The problem is “*how”* the Invisible Hand does really work; in the end, we should (and could) explain the “mechanism” and its reproductive feedback on the agents’ minds and behaviors.

In Smith’s view of the “Invisible Hand”:

(1) there are intentions and intentional behavior;
(2) some unintended and unaware (long term or complex) effect emerges from this behavior;
(2) but that effect is not just an effect, it is an *end* we pursue, i.e., its orients and controls – in some way – our behavior: we “necessarily operate for” (Smith, ibid.) that result.

Now:

– what does it mean and how is it possible that we promote with our action, we in a sense *pursue* something that is not an intention of ours; that the behavior of an intentional and planning agent be goal-oriented, finalistic, without being intentional?
– in which sense the unintentional effect of our behavior is an “end”?

The real problem is to understand how not only such process coexists with an intentional behavior but also exploits it (Castelfranchi, 2001).

Thus special attention should be devoted not only to the “emergent” bottom-up processes but also to the “immergent” ones: the top-down feedback from emergent phenomena to the agent control-system via learning or through understanding and intending (Conte et al., 2007).

In particular we have to identify which of the macro-level phenomena is or *has to be* mentally represented, understood, and even intended in order to reproduce itself and be effective (as it happens with norms), and to discriminate those that are unintended and blind, and presuppose some form of alienation (like social functions or institutional powers). What we have to explain is also how the Invisible Hand and spontaneous (self-organizing) social order are not so spontaneous and disinterested or optimal for the involved people but do systematically favor powerful agents. What is needed is a criticism to von Hayek’s theory (or vulgate) about the spontaneous social order as the best *possible* outcome: the often implicit assumption that *an understanding of the social dynamics, deliberate planning, and intentional pursuuit of non-individual outcomes could never achieve better results*.

How much the epistemic and motivational representations that regulate our intentional conduct are *shaped by* the macro sociological, economic, anthropological, political levels? How the former *are functional to* the latters, not just mere complex effects and consequences?

That is: how could the Spontaneous Order not just *emerge* from our autonomous acts but *maintain and reproduce* itself without actively influencing and reproducing those acts? Which – however – are due to our cognitive representations and processes. Thus it has to shape and reproduce those cognitive mechanisms. *The Invisible Hand works also through and on our minds, by manipulating our mental devices in order to bring out the appropriate (not understood and unintended) outcomes.*

In fact, the problem is not just how a given *equilibrium* (like in simple Games) or *coherence* is achieved and some stable *order* emerges. In order to have a “social order” or an “institution” spontaneous emergence and equilibrium are not enough. They must be “functional,” that is self-reproducing by a causal loop.

### THE “*COGNITIVE MEDIATORS”* OF SOCIAL PHENOMENA

Social phenomena are due to the agents’ behaviors, but… the agents’ behaviors are due the *mental mechanisms* controlling and (re)producing them.

For example: Our Social Power lies in, consists of, others’ *Goals* & *Beliefs*! How do they evaluate us and accept to depend on us. That’s why we need Mind-Reading! Not only for adjusting ourselves to the others’ interference, but for manipulating and exploiting the others or for helping or punishing them.

Social and cultural phenomena cannot be deeply accounted for without explaining how they work *through the individual agents’ minds* (the mental “counterparts” or “mediators” of social phenomena).

Does this mean that social actors fully understand what they do/construct? No, not necessarily.

That’s why we use the term: “mediators”: because they are the mental ingredients necessary for producing that social phenomenon or structure without *(necessarily) being the mental representation (understanding or intending) of the social phenomena produced by the behaviors that they determine.*

So, I play and reproduce a “social function” (of father, consumer, the witness of a promise, “public opinion,” the follower of a leader, etc.) without necessarily understanding it, but with something specific, corresponding, in my head.

As we said, the problem is social functions impinge not only on our habits and automatic or ritual behaviors, but on our deliberated and intentional actions. Charging only the non-intentional, non- deliberate behaviors with those functional aspects is a simplistic solution: according to such a view, role-playing would just be implemented in “habituses” (Bourdieu and Wacquant, 1992). Thus, when a social actor is consciously deliberating and planning, he would not play a social role, he would be “free.” I disagree with such a solution. Social actors play social roles and accomplish their social functions also through their deliberate, intentional actions, however they do so not deliberately. This is precisely the problem to be addressed; and it requires a sophisticated model of intentions. We are back to the issues of (C;Two Teleologies Impinging on Human Behavior).

*What is the relationship existing between the social system’s goals and the goals internal to its members, which directly regulate their actions*?

Are social actors able to understand and represent explicitly in their minds the social system’s goals? Or are the goals of the social system simply a projection of the goals of (some of) its members? Or, do the members’ goals and plans happen to happily coincide with those of the social system? In other terms: do we intends all the goals we pursue?

Functions establish and maintain themselves thanks to and through agents’ mental representations but not as mental representations: i.e., without being known or at least intended.

## COMPUTATIONAL SCIENCE FOR RECONCILING “EMERGENCE” WITH “COGNITION”

However, “necessary” doesn’t mean “sufficient”: Mind is not enough. For “explaining” what is happening at the societal and collective layers we have to model the mind of the actors, but this is insufficient. The “individualistic plus cognitive” approach – even if complemented with “collective intentionality,” “joint action,” “we intend,” etc. – is not sufficient for a social theory and for modeling social processes. Social actors do *not* understand, negotiate, and plan all their collective behavior and cooperative activity. Society is not “team work.”

This is the real challenge not only for the behavioral and cognitive sciences but for multi-agent systems and Social AI, and computer-supported societies: *Reconciling Emergence with Cognition.* Emergence and cognition are not incompatible with one another; neither are they two alternative approaches to intelligence and cooperation.

On the one hand, cognition has to be conceived as a level of emergence (from sub-symbolic to symbolic; from objective to subjective; from implicit to explicit).

On the other side, emergent and unaware functional social phenomena (ex. emergent cooperation, and swarm intelligence) should not be modeled only among sub-cognitive agents (Steels, 1990; Mataric, 1992), but also among intelligent agents. In fact, for a theory of cooperation and society among intelligent agents – as we said – *mind is not enough,* and cognition cannot dominate and exhaust social complexity (on that Hayek is right; Hayek, 1967).

This is why a crucial revolution in the behavioral sciences is and will be “computational modeling,” with its radical “operational” approach. There is no alternative to this, especially if one has to model at the same time the process at a given micro-layer and the processes at the macro-layer, and also the emergent (bottom-up) and the immergent (top-down) feedbacks, and how all this works.

We need a computational modeling of cognitive representations and manipulation (processing; Cognitive Mechanisms Producing and Controlling Our Behavior) and a computational modeling of their neural implementation and of brain very complex dynamics (The Neural Implementation of Psychological Representations and Processes). The same holds at the social level.

### THE *THEORETICAL* MISSION OF SOCIAL SIMULATION

Agent-based computer simulation of social phenomena is the crucial (revolutionary) challenge for the future of behavioral sciences. But why is it so?

As we said, the micro-macro link is the *foundational* issue of the behavioral sciences: they should investigate the relation between cognition and individual behavior, on the one hand, and social self-organizing phenomena or complex structures, organizations, and institutional actions and entities^6^ on the other hand. This is the main mission of the social sciences, what they have to “explain,” diachronically and synchronically.

No approaches or models for studying this complex phenomenon and eventually understanding its (causal) mechanisms are better than agent-based computer simulation. It is the only approach able to model *at the same time* different layers of processing and their top-down and bottom-up feedbacks and circularity. We can model more or less complex minds (with goals, beliefs, reasoning, decisions, etc., but also emotions, reactions, biases, and perception, learning, etc.) and interaction, dependence networks, group activity, organization, cooperation and competition, norms, roles. And we can observe the internal and external dynamics.

Moreover computer implementation of models provide us a formal validation of the theory predictions, and new experimental data (by simulation).

## CONCLUDING REMARKS

What is the correct relation between social and collective human behaviors and the individual mind, and between mind and brain? The answer is: *a well-conceived reductionism,* preserving different (interconnected) ontological layers with their vocabulary (like in chemistry for the notion of “valence” or of “acid”).

### A LAYERED SCIENCE FOR A LAYERED WORLD

Nature (and, in nature, society) has *different levels of complexity and organization*, with the emergence of macro-level entities, phenomena and laws, grounded on the entities, properties and mechanisms of the lower layer (micro).

“Reductionism” should not be the “elimination” of the entities, notions, dynamics of a given macro level, considered superfluous once it is explained in terms of their micro-entities. “Reductionism” should be “re-conduction”: bringing back and grounding the macro-dynamics on the underlying ones. The theories of the macro layer should be not only *compatible* (non contradictory) with the laws of the micro one; they have to be grounded and derivable. Otherwise they are wrong.

Consider for simplicity the following layers of complexity: let’s ground all on physics (particles, atoms, forces, etc.); on top of physics, let’s put chemistry, then biology grounded on organic chemistry, then neuroscience, then psychology, then social sciences (economics, sociology, anthropology, politics).

Biology has to be explained in biochemical terms, but we cannot eliminate the notion of “cells” with their new properties and laws, although we have to biochemically know how they “work”^7^.

In the same vein, social and collective behavior is due to the conduct of individual actors; but individual action is due to mental representations and processes; therefore the principles of social sciences should be grounded in the underlying mental and behavioral phenomena and laws. However after such a re-conduction is made, we cannot do without such notions as crowd, market, inflation, government, etc.

Science has for example re-conducted chemical “valence” (introduced much before atomic modeling) to atomic properties: particles, their electric charge, etc. To have explained “valence” and how it works doesn’t make this notion useless; and we couldn’t put aside notions like “acid,” “basis,” “chemical bonds” although we have a full grounding and understanding of them in atomic terms. The Phlogiston theory has been eliminated, because there was no possible confirmation of the hypothesized processes at the supporting/implementing layer.

Exactly in the same way we have to re-conduct mental representations, functions, and processing to the body and its neural mechanisms and structures; they are just material, informational entities^8^; emergent *functions* of their ground, described in informational/functional terms. If it is not possible to bring them back to their sub-stratum, they are inexistent (like phlogiston); but if they are brought back to their underlying micro-processes, they will not be redundant and eliminable. The psychological notions should be preserved for understanding and explaining “what the brain is *doing*”: perceiving, memorizing, retrieving, deciding, pursuing, and so on; at its macro-functional level of activity.

Neural correlates cannot be the right *vocabulary* for explaining human behaviors, just because they are at a micro-level and do not still represent and discriminate the complex “patterns” and their properties and functions (not of their sub-components) at the cognitive and motivational macro-level of working. When we will have the real neural representation of a complex object like a “motivating goal,” or an “altruistic intention,” or of real “trust attitude” (The Neural Implementation of Psychological Representations and Processes), or a “complex emotion with its appraisal components” like envy, we will have a quasi-complete explanation of it (see previous note), but we will not renounce to that psychological vocabulary; since it holds and works at the functional/informational macro layer. Also because, there are other properties of that entity that are due not to its micro-implementation and mental representation, but to its functions and relations at the macro anthropological, sociological, economic level. A table is a “table,” functionally and practically speaking, although it is just a cluster of molecules of a given substance; however, at certain level of use its analysis in physical and material terms is fully irrelevant.

More in general: there are no alternatives to the need for *reading* and *understanding* body in terms of functions, not just in terms of “simple” matter and its physico-chemical processes description.

We look at the kidney as a “filter,” at glands in terms of “secretion.” Otherwise we do not understand what they do, that is, what they are; which is the sense of the physico-chemical processes that we are describing.

The same obviously holds for our brain (just a body organ). Brain anatomy must be a “political geography,” not a “geography” of physical objects/structure: it has to localize the areas of given psychological functions. And brain physiology (activity) – to be understood – requires to be read in terms of active psychic processes. “Mind” is just a functional notion: the high level function of neural activity and patterns; and given its *emergent, functional, informational, semiotic-representational* (and even *institutional*) nature is not “reducible” to brain processes, that just provide its material implementation.

We need a micro-macro theory, a specification of the underlying entities and processes producing given phenomena at the superordinate layer. This is what we call – in strict sense – *Science of “mechanisms.”*

### THE NEED FOR COMPUTATIONAL MODELING

I claim that there is no alternative to computer modeling. We have to provide not just mathematical or formal models but computational ones, if we want to model the proximate causes of a given phenomenon, and its superficial dynamics; the *underlying “mechanisms” that determine those behaviors*. We also need “synthetic” modeling, that is, the material construction of the modeled entity to show how it actually produces the predicted behaviors/effects in interaction with the environment.

As rightly pointed out by Shieber (2004): “The whole thinking process is still rather mysterious to us, but I believe the attempt to make a thinking machine *will help us greatly in finding out how we think ourselves*.”

*Computational/synthetic modeling* will be pervasive. It will model any hidden mechanism and “dynamics”: from chemical reactions, to DNA, from evolution to psychological mechanisms, to social, economic, historical phenomena. This is the message and the *gift* that ICT and in particular AI has to give to science.

Computational modeling will provide not only “models” and conceptual instruments for the theory, but also *experimental platforms*, new empirical data obtained through simulation, and new hypotheses and predictions. Some experiments will be made possible, which are impossible in “nature,” either for practical, social, historical, or moral reasons (demography, urbanistic, etc.) or for the natural inseparability of some distinguishable mechanisms (for example motivational and emotional mechanisms). This will be crucial both for modeling both proximate causes and diachronic, evolutionary causes.

Computer simulation of neural, cognitive, social mechanisms and dynamics, obviously *is not the only method* for identifying the proximate causes, but the most promising method for (i) their fully *procedural* and *formal* characterization (ii) with the additional advantage of “running” the postulated dynamics and seeing their results (conform or not to predictions), and (iii) to conduct a new precious kind of experiments, in particular useful for complexity and emergent effects. The most promising method/tool, especially for modeling “processes” not just static features (physiology not just anatomy) (iv) at different layers, but interacting; including also the bottom-up and the top-down effects, and the resulting dynamics. It is the only approach able to deal in an integrated way with all these mechanisms.

Moreover, computers are a fundamental device for intelligently *collecting and analyzing relevant data* [from the web, for example “Big Data,” and from human behavior in natural conditions (traffic, investments, migration, etc.)]. Also in the sense that major scientific discoveries will be made by computers (able to manage Big Data, to demonstrate theorems, to interpret the laws and mechanisms of that data), but also in the sense of “human” traditional science supported by computational instruments.

In sum, in a few years, science will be “computational”; otherwise it will not be.

### “MORE GEOMETRICO DEMOSTRATA”

To be more explicit about psychology status: It is unbelievable that after more than half a century the critical remarks of Wittgenstein on psychology be still valid: “*The confusion and barrenness of psychology* is not to be explained by calling it a “young science”; its state is not comparable with that of physics, for instance, in its beginnings. (…) For *in psychology there are experimental methods and conceptual confusion*. (…) The existence of the experimental method makes us think we have the means of solving the problems that trouble us; though problem and method pass one another by” (Ludwig Wittgenstein *Investigations* PII p. 232).

In my view, Psychology is one of the few sciences that do not officially have a clearly separated theoretical domain, with its university chairs, conferences, curriculum, … like for physics, biology, economics, … without any direct experimental activity (in case taking into account and *explaining* the result of their “experimental” discipline; and anticipating of half a century the empirical results, like for Einstein theories).

Psychology – probably because of its “guilty” origin from philosophy, and the consequent inferiority complex to the “hard sciences” – has repressed its theoretical and analytical impulses.

Philosophy is in a sense a party to this somewhat phobic attitude of psychology, because it considers the analytical, formal, theoretical work as a prerogative of its own. However, philosophical contributions, though welcome, cannot replace the theoretical and analytical work that must be internal to psychology.

It is not a matter of “experimental philosophy,” it is a matter of “theoretical psychology” (Cognitive Science sometime plays such a role).

We need to have psychological states “more geometrico demostrata.”

### JUST STATISTICAL LAWS AND CONSTRUCTS, AND PROBABILITY?

However, let us conclude with a query about next future, by following not just optimism of will but pessimism of reason:

Will this analytical and “cognitive mediated” view of social phenomena and dynamics, and of computational Agent-based modeling (we hope for) win?

Not so sure at all: we will attend a short cut of statistics, the impressive power of Big Data, correlations, probability, … An already very robust trend. The title of Mayer-Schonberger & Cikier’ book is “Big Data: A Revolution That Will Transform How We Live, Work, and Think” and I think that they are absolutely right; but this revolution will be insufficient and even deviating if it will just empower our “predition” capabilities, and will not ground new *theoretical understanding* of the mechanisms and causal processes underlying society and cognition. I worry about Anderson’s profecy: “The End of Theory: The Data Deluge Makes the Scientific Method Obsolete”; prophecy that of course begin to be based on Big Data! And I care more about scientific aim and frame than about scientific “methods” (See also Anderson, 2008; Harford, 2014).

We are witnessing a growing trend of *predicting without understanding*, without modeling the proximate causes.

Does God play dice?

## Conflict of Interest Statement

The author declares that the research was conducted in the absence of any commercial or financial relationships that could be construed as a potential conflict of interest.

## Footnotes

2 *Beneficium non in eo quod fit aut datur consistit, sed in ipso dantis aut facientis animo*: a benefit consists not in what is done or given, but in the intention of the giver or doer *(Seneca, De Beneficiis Libro I, 6).*

3 However, also consider other bases of mental entities and process, in term of externalization and distributed cognition, or in radically institutional, conventional terms (as we have just said).

4 For a deep philosophical and critical discussion of teleologies and the relation with causal explanation in natural sciences, see Larry Wright fundamental work (Wright, 1976). A remarkable attempt to deal with these problems also is Ruth Millikan’s work.

5 See for example (with a more traditional approach) Sawyer, 2003 or Prietula etal., 1998; also related with agent-based simulation (Computational Science for Reconciling “Emergence” with “Cognition”). For interdisciplinary and integration based view close to our position, see also (Dale etal., 2013). Although in our perspective goal-directed behavior and intended results and self-organization and spontaneous social order are two complementary and interacting faces of sociality and social theory. We do not think that dynamical system theory is the framework for integrating human interaction “into a broader account.” Also because the problem is not the coordination of motor, expressive, and linguistic “inter-action,” but of social conventions, scripts, institutions, and collective self-organizing “order.” We are more in agreement with the previous Dare’s claim about the need for a “pluralistic” approach in Cognitive Science.

6 The two faces of “social order”: the “spontaneous” one and the organized or at least institutionalized one.

7 To say nothing of the “evolutionary theory”: a completely new foundation.

8 However, see note 13.
